# Examining the association of family environment and children emotional/behavioral difficulties in the relationship between parental anxiety and internet addiction in youth

**DOI:** 10.3389/fpsyt.2024.1341556

**Published:** 2024-06-03

**Authors:** Yuxin Wang, Keyin Zhou, Yang Wang, Jing Zhang, Yuanchen Xie, Xin Wang, Wenyi Yang, Xiyan Zhang, Jie Yang, Fei Wang

**Affiliations:** ^1^ Department of Psychiatry, Affiliated Brain Hospital of Nanjing Medical University, Nanjing, Liaoning, China; ^2^ Fourth School of Clinical Medicine, Nanjing Medical Universtiy, Nanjing, Jiangsu, China; ^3^ Institute of Brain Functional Imaging, Nanjing Medical University, Nanjing, Jiangsu, China; ^4^ Department of Child and Adolescent Health Promotion, Jiangsu Provincial Center for Disease Control and Prevention, Nanjing, Jiangsu, China; ^5^ Department of Mental Health, School of Public Health, Nanjing Medical University, Nanjing, Jiangsu, China

**Keywords:** internet addiction, parental anxiety, family environment, emotional, teenager

## Abstract

**Introduction:**

Associations between parental anxiety and adolescent internet addiction have been documented in the literature; however, few studies have analyzed the role of the family environment in this relationship. This study aims to explore the relationship between parental anxiety and adolescent internet addiction while also investigating the indirect relationships involving multiple dimensions of the family environment and child emotional behavior issues.

**Methods:**

Surveys were conducted among 6,296 parent-child pairs. We administered SDQ, CIAS-R, and FES-CV to assess adolescents’ issues and internet addiction, and evaluate family environment. Additionally, parents completed GAD-7 to assess parental anxiety levels.Results: Correlation analysis revealed that the family environment and adolescent emotional behavior issues played an indirect relationship in the link between parental anxiety and internet addiction.

**Discussion:**

The findings emphasize the importance of addressing parental anxiety and fostering a positive family environment as effective measures to alleviate adolescent emotional behavior problems and reduce the risk of internet addiction.

## 1 Introduction

Adolescents’ engagement in online activities plays a vital role in their leisure, entertainment, and social interactions. However, internet addiction has become one of the most prevalent maladaptive behaviors worldwide, with internet gaming disorder being recognized in the DSM-5 (1). These behaviors pose substantial threats to the psychological and physical well-being of adolescents and may impact their future developmental paths (2, 3). Epidemiological studies, primarily focusing on male patients, have linked internet addiction to psychosocial challenges, psychopathological disorders, compromised physical health, and diminished quality of life (4). Furthermore, the COVID-19 pandemic has led to significant shifts in the lifestyles and learning routines of adolescents. Spending more time at home has resulted in increased usage of electronic devices. For adolescents with weaker self-management abilities, this newfound opportunity may be utilized for recreational activities, potentially reinforcing their reliance on the internet (5, 6). These changes may intensify adolescents’ propensity for internet usage, rendering them more vulnerable to the perils of internet addiction. Consequently, internet-related issues among adolescents have become a prominent societal concern, carrying significant implications for families and society as a whole (7). Hence, conducting a comprehensive investigation into the potential predictive factors of adolescent internet addiction is of paramount importance to devise effective intervention measures.

Numerous studies have identified certain predictive factors for internet addiction, such as sleep quality (8), psychopathological disorders (9), and social support (10). Several studies have established a link between parental emotional difficulties (including anxiety, depression, behavioral and psychological controls), and internet addiction (11, 12), but the impact of family environment remains worthy of further study. Based on the family systems theory and ecological systems theory, this study aims to explore the relationship between parental anxiety and adolescent internet addiction, while also examining the role of multidimensional family environment (including intimacy, conflict, achievement orientation, cultural orientation, recreational orientation, organization, and control), as well as child emotional and behavioral issues in this association.

### 1.1 Parental anxiety and internet addiction

In recent years, there has been widespread concern about the parental anxiety and its adverse effects on the mental health of adolescents. Research findings show that approximately 66.8% of parents in China experience elevated levels of anxiety (13). Studies underscore the possibility that parental anxiety may lead to behavioral and psychological control, deterioration in parent-child relationships, and neglect, particularly as these factors are closely intertwined with smartphone and internet addiction among adolescents (14–18). Furthermore, anxious parents may manifest specific behaviors related to their emotional state (for example, excessive worry, nervousness, and manifestation of negative emotions), this process may lead children to adopt similar ways of coping with stress and handling emotions as their parents (19). It could be a key mechanism contributing to adolescent addictive behaviors, such as internet addiction. While the exact mechanisms are not fully understood, we believe that parental anxiety may influence the family atmosphere and shape children’s emotional coping mechanisms through various pathways. Firstly, anxious parents may create a tense and uneasy atmosphere within the family, which could hinder communication among family members and escalate internal tensions and conflicts (20). Secondly, anxious parents may exhibit excessive worry, nervousness, and negative emotions, which could be transmitted to their children, impacting their emotional state and regulation abilities (21). For instance, when parents display anxiety and tension, children may experience feelings of unease and fear, and may adopt similar emotional coping strategies. Additionally, anxious parents may tend to employ overly protective or controlling parenting styles, which could limit children’s autonomy and self-development, impeding their ability to effectively manage emotions and cope with stress (22). Therefore, by researching the potential correlation between parental anxiety and adolescent “addictive behaviors,” we can gain a deeper understanding of this phenomenon and provide more targeted recommendations for intervention measures.

### 1.2 Family environment and internet addiction

Regarding family factors, family relationships have been proven to be a key mediating factor in transmitting parents’ emotional problems to children’s emotional and behavioral issues. Academic research emphasizes that family and parenting environments, as a dynamic system, impact various aspects of adolescents (23–25). Internet addiction is closely related to the internalizing and externalizing problems in adolescents. Firstly, internet addiction is often categorized as an externalizing problem, thus it is likely influenced by parental anxiety (26). On the other hand, internalizing problems involve individual’s internal emotions and psychological states, where internet addiction may serve as a coping mechanism to escape or alleviate negative emotions (27, 28). However, previous studies often discuss the family environment as a whole or focus on specific aspects of it (29). The ecological systems theory and family systems theory both emphasize the importance of the environment in individual development. The ecological systems theory focuses on the interaction between individuals and their surrounding environment, highlighting the influence of environmental factors on individuals, and considers the family as one important ecological system. Within this framework, individual behavior, emotions, and development are influenced by the family environment, and the interactions and dynamics among family members have an impact on individual growth (30, 31). On the other hand, family systems theory views the family as a holistic system, emphasizing the interactions and interrelations among family members. The behavior, emotions, and relationship quality of parents affect the stability and functioning of the family system. Parental anxiety may disrupt the balance of the family system. Anxious parents may exhibit more tension and negative emotions, which can affect the interactions and emotional connections among family members. They may demonstrate excessive control, detachment, or conflict, leading to a tense and unstable family atmosphere, which could negatively impact the emotions and behaviors of adolescents (32, 33). If the family environment is characterized by tension, instability, or other negative features, it may have adverse effects on a child’s development. Parental anxiety can manifest within the family atmosphere, influencing the emotions and behaviors of the child (33). During the COVID-19 pandemic, one study focused on investigating the overall impact of the family environment on children’s diet and nutritional status (34). In contrast, research examining the role of family intimacy in collaborative esports using the Switch device concentrated on the dimension of family relationship intimacy (35), aiming to understand its specific effects on the gaming experience. This type of study, which delves into specific family dimensions, holds significant importance as it helps us comprehend how a particular family factor can have a comprehensive impact on individual behavior and health in diverse contexts. By exploring multiple dimensions of the family environment, this paper aims to precisely grasp the mechanisms through which the family influences individuals in specific situations, providing more targeted recommendations and strategies for future family-based interventions. For example, if research reveals that the way a family resolves conflicts is related to the psychological well-being of adolescents, intervention measures can focus on providing training in conflict resolution skills to help family members effectively address conflicts and stressors. Similarly, if it is found that family cohesion is associated with adolescents’ adaptability, interventions may involve strengthening communication and support networks among family members to enhance adolescents’ emotional stability and social adaptation skills. Therefore, through targeted intervention measures, different needs within the family can be better addressed, leading to specific improvements in family dynamics and individual health. This study employs a multidimensional conceptualization of the family environment, incorporating multiple subscales to comprehensively assess various aspects within the family. These subscales cover dimensions such as cohesion, emotional expression, conflict resolution, individual independence, achievement orientation, cultural interest, recreational activities, moral-religious orientation, organization, and control among family members. By meticulously examining the impact of each family environmental construct, we aim to provide a more profound and comprehensive understanding of the relationship between family factors and adolescent internet addiction. For example, in families characterized by low intimacy and high conflict, adolescents may opt for online social interactions as an alternative to communicating with their parents, resulting in spending more time on the internet. In families characterized by low intimacy and high conflict, adolescents often opt for online social interactions as a substitute for communication with their parents, consequently spending more time on the internet. In such scenarios, the social circle of adolescents plays a crucial role in the development of their addictive behaviors. Research indicates that associating with peers and friends who exhibit addictive behaviors significantly increases the risk of adolescents developing their own addictive tendencies (36). These adolescents are more likely to encounter peers already addicted to the internet, and they are influenced by the behaviors and attitudes of their peers (37). They may emulate their peers’ behaviors or be influenced by them, thereby exacerbating their own internet usage and addiction. Additionally, with low parent-child intimacy and frequent family conflicts, parents often lack adequate guidance in helping their children establish healthy friendships. This contributes to the difficulty in correcting these adolescents’ poor socializing tendencies. Similarly, in households with limited engagement in recreational activities, where parents spend less time playing with their children, adolescents may be more inclined to use the internet to fulfill their entertainment needs (38). In such an environment, if the family lacks organization and rules are unclear, adolescents may feel a lack of guidance and structure (39), thus more likely to become immersed in the online world to seek self-organization and rules. Simultaneously, we will also explore the relationship between family environment and adolescent internet addiction in the dimensions of cohesion (40), conflict resolution (41), achievement orientation (42), cultural interest (43), and recreational activities (44). Therefore, this paper also aims to investigate the relationship between family environment and adolescent internet addiction across these dimensions, seeking to identify family environmental factors that predict adolescent internet addiction.

### 1.3 Adolescent emotional and behavioral issues and internet addiction

Previous studies have demonstrated a strong relationship between internet addiction and the mental well-being of adolescents. For instance, internet addiction may increase susceptibility to depression and anxiety (45), negatively impact academic performance, physical exercise, and sleep, and even have a moderate correlation with Attention Deficit Hyperactivity Disorder (ADHD) (46). Adolescents facing emotional challenges, such as academic pressure, family issues, and social challenges, may resort to excessive internet use to escape reality and alleviate emotional distress (47). Prolonged immersion in the online world may also lead to social isolation in adolescents, leading to a lack of emotional and interpersonal skills in facing real-life situations, thereby increasing the risk of emotional problems (48, 49). Most previous research has primarily examined the relationship between internet addiction and the mental health of adolescents, however, this study will assess five aspects, including emotional symptoms, conduct problems, hyperactivity, peer problems, and prosocial behavior, to explore the connection between adolescent emotions and behaviors and internet addiction from the perspectives of internalizing and externalizing issues.

### 1.4 Parental anxiety, family environment, and adolescent emotional and behavioral issues

Regarding individual factors, numerous studies have confirmed that parental anxiety and depression may significantly influence the anxiety and depression of adolescents, potentially fostering internet addiction. Parents with anxiety may excessively focus on negative events (50), leading to intensified parenting stress and resulting in internalizing and externalizing problems in their children (51). The family environment is inseparable from emotional and behavioral challenges in adolescents. Families with mental health issues often exhibit weakened cohesion, diminished adaptability, and increased conflict, making children more susceptible to disturbances in psychosocial functioning and stress response systems (52, 53). The literature on the relationship between parental parenting styles and family environment suggests that adolescents with higher levels of parental anxiety may experience increased levels of family conflict. Additionally, findings indicate that adolescents with higher levels of anxiety tend to perceive their parents as more isolated within the family environment, excessively concerned with others’ opinions, and feel ashamed of their own perceived inadequacies (54). While previous studies often focused solely on either parental individual factors or family environmental factors, our research simultaneously considered both of these influencing factors, thoroughly exploring their associations with adolescent emotional and behavioral issues.Additionally, we conducted meticulous analysis to thoroughly study the relationships among these factors. Although cross-sectional data cannot provide causal evidence, our emphasis is on elucidating the correlations between these factors. This comprehensive approach aids in better identifying factors that are more predictive of the severity of internet addiction.

### 1.5 The current study

In summary, the escalating recognition of the detrimental impact of parental anxiety on adolescent mental health parallels the growing global concern over the increasing prevalence of internet addiction among this demographic. However, a research gap exists, notably the lack of a detailed categorization of the family environment into different dimensions to explore their relationships with other factors separately. Additionally, previous studies using small samples may have compromised the credibility and statistical significance of the findings. To address this gap, we propose the following specific research questions and hypotheses. Hypothesis one suggests that family environment variables (including closeness, conflict, achievement orientation, cultural orientation, recreational orientation, organization, and control) play an indirect role in the impact of parental anxiety on adolescent internet addiction. Hypothesis two anticipates that adolescent emotional and behavioral issues will generate an indirect relationship between family environment variables and internet addiction. Through the study of these factors, our aim is to explore the relationship between parental anxiety, family environment, adolescent emotional and behavioral issues, and internet addiction. It is hoped that this research will provide support for the establishment of intervention measures to address adolescent internet addiction, further improve family environments, and enhance the overall well-being of adolescents.

## 2 Method

### 2.1 Participants

We conducted a questionnaire survey among students aged 10-18 and their parents in Taizhou, Yixing, and Sheyang in Jiangsu Province, China. Prior to the survey, we obtained informed consent from all participants. To ensure representation, we selected 1-2 primary schools, 1-2 middle schools, and 1-2 high schools in each location, using a cluster stratified random sampling method. Utilizing a convenient and efficient online self-monitoring system, we assessed the psychological health status of individual students and the overall well-being of the student community through self-assessment questionnaires completed by both students and parents. We collected a total of 6,296 valid questionnaires, among adolescents, there are 2,884 females and 3,412 males, and among parents, there are 5,294 mothers and 1,002 fathers.

### 2.2 Measures

We administered the Strengths and Difficulties Questionnaire (SDQ), the Revised Chen Internet Addiction Scale (CIAS-R), and the Family Environment Scale-Chinese Version (FES-CV) to assess adolescents’ emotional and behavioral issues, measure the extent of internet addiction, and evaluate the family environment, respectively. Additionally, for parents, we utilized the Generalized Anxiety Disorder-7 scale (GAD-7) to assess the level of parental anxiety.

#### 2.2.1 Strengths and difficulties questionnaire

SDQ was a self-report questionnaire with 25 items that was developed by Goodman (55) with the aim of measuring the mental strengths manifested in children and the difficulties faced by the children, we administered this questionnaire as a self-report for adolescents. It had five subscales, including subscales of emotional symptoms (e.g., Many worries, often seems worried), conduct problems (e.g., Often lies or cheats), hyperactivity (e.g., Thinking things out before acting), peer problems (e.g., Rather solitary, tends to play alone), and prosocial scale (e.g., Kind to younger children). Participants responded to each item via a three-point scale (1 = “not true,” 2 = “somewhat true,” and 3 = “certainly true”), and high scores indicate high levels of mental strengths and difficulties. The Chinese version of the SDQ had good reliability and validity (56, 57), and the Cronbach’s alpha of the total four subscales was 0.71. In calculating adolescent emotional and behavioral issues, we utilized the total scores of emotional symptoms, conduct problems, hyperactivity symptoms, and peer problems. The total difficulty score, with a range from 0 to 40, was categorized as follows: scores equal to or less than 13 were considered within the normal range, scores from 14 to 16 were categorized as borderline, and scores ranging from 17 to 40 were considered abnormal.

#### 2.2.2 The revised Chen internet addiction scale

CIAS-R was a modified version of the Chinese Internet Addiction Scale (CIAS) developed by Taiwanese scholars (57), including Chen Shu-Hui. It consisted of 26 items grouped into “Core Symptoms of Internet Addiction” and “Internet Addiction-Related Problems.” Adolescent participants completed self-reports of the CIAS-R. The “Core Symptoms of Internet Addiction” factor comprised three sub-factors: Compulsive Use of the Internet, Withdrawal Symptoms of Internet Addiction, Tolerance Symptoms of Internet Addiction. The “Internet Addiction-Related Problems” factor included two sub-factors: Interpersonal and Health-Related Problems of Internet Addiction, Time Management Problems. Participants rate each item on a 4-point scale (1 = “Not at all,” 2 = “Slightly,” 3 = “Moderately,” and 4 = “Severely”). The score for each factor was the sum of the scores of the items it contains. In the calculation, we summed up the scores of these two factors to assess the severity of internet addiction, higher scores indicated a more severe level of Internet addiction. Adopting the recommended cutoffs for adolescents (58), respondents with CIAS-R scores of 64 or above were classified as Internet addicted. Cronbach’s alpha of the total score of CIAS-R was reported to range from 0.90 [10] to 0.95 (59) in university students, and was 0.97 in the present study.

#### 2.2.3 Family environment scale-Chinese version

FES-CV was a self-report questionnaire introduced and revised by Fei Lipeng et al. in 1991 (60), adapted from the FES developed by Moss et al. in 1981 (61), this was a self-report questionnaire for adolescents. It aimed to assess 10 different family environmental characteristics and explore the impact of the family on individuals. This questionnaire encompassed ten subscales aimed at evaluating various relational characteristics within the family environment. These subscales included Cohesion, which measured the commitment and support among family members; Expressiveness, which gauged the encouragement of emotional expression within the family; Conflict, which assessed the openness of anger expression and conflict resolution; Independence, which examined the level of esteem, self-confidence, and independence among family members; Achievement Orientation, which appraised the family’s perspective on achievement and competition in general activities; Intellectual-Cultural Orientation, which indicated the family’s interest in political, intellectual, and cultural pursuits; Active-Recreational Orientation, which assessed family involvement in recreational activities; Moral-Religious Orientation, which measured the emphasis on ethnicity, religion, and values among family members; Organization, which evaluated the planning and responsibility allocation for family activities; and Control, which determined the extent to which rules and procedures were used by family members to structure their lives (62). The scale consisted of 90 true/false items divided into 10 subscales. However, it was found that the subscales measuring Independence, Moral-Religious Emphasis, and Expressiveness demonstrated relatively poor internal consistency reliability, possibly due to their content being less suitable for Chinese culture. Therefore, for the purpose of this screening, 7 subscales (Closeness, Conflict, Achievement Orientation, Cultural Orientation, Recreational Orientation, Organization, and Control) with better reliability and validity were selected, resulting in a total of 63 true/false items utilized. The FES-CV demonstrated good reliability and validity, except for three subscales: expressiveness, independence, and moral-religious orientation. These subscales were excluded from our study; thus, a total of seven subscales were utilized. The Cronbach’s α coefficients for the cohesion, conflict, intellectual-cultural, organization, achievement, active-recreation, and control subscales were 0.813, 0.807, 0.798, 0.764, 0.712, 0.726, and 0.708, respectively, indicating satisfactory internal consistency reliability for the measurement instrument (63).

#### 2.2.4 Generalized anxiety disorder-7 scale

GAD-7 was a self-report screening tool developed by Stanley Rachman (64) with the aim of measuring parent’s anxiety symptoms, this was also the sole self-report applied to parents. This scale comprised 7 items (worry, tension, irritability, muscle pain, fatigue, difficulty concentrating, and irritability), each assessing various aspects of anxiety symptoms. Participants rated each item on a four-point scale ranging from “0 =not experienced at all” to “3 =experienced almost every day”.The scale had a total score of 21 points, achieved by adding up the scores from each item given by the test taker, and a higher score indicates a greater level of anxiety.The assessment criteria for anxiety were as follows: A score of 0-4 indicates no symptoms of anxiety; 5-9 points suggest possible mild anxiety symptoms; 10-13 points may indicate moderate anxiety symptoms; 14-18 points could suggest moderately severe anxiety symptoms; and a score of 19-21 may indicate severe anxiety symptoms.The Chinese version of GAD-7 was widely used in research and clinical practice, and had a Cronbachs alpha of 0.89.

### 2.3 Data analysis

The current study employed a series of well-defined steps for data analysis. Firstly, a total of 10,232 pairs of parents and children completed the questionnaire survey. To meticulously clean the data, the author implemented rigorous measures to exclude outliers. During this process, it was noted that some participants provided answers in demographic questions that did not match the given options. Consequently, these outliers were removed by the author to ensure the accuracy and reliability of the data. Subsequently, data from participants who did not complete questions related to parental anxiety, family environment, adolescent emotional behavior, and teenage internet addiction were further excluded. The final dataset comprised 6,296 valid responses. A descriptive analysis of demographic characteristics was then conducted, wherein the participants were grouped based on the levels of parental anxiety. The family environment was categorized into seven dimensions, and a direct model was employed to explore the intricate relationships between all variables. For the purpose of conducting indirect association analysis, one independent variable (parental anxiety), one dependent variable (adolescent internet addiction), and two intermediary variables (family environment and adolescent emotional and behavioral difficulties) were utilized. We categorized parental anxiety into three levels and conducted analysis of variance (ANOVA) among these levels. Additionally, we employed Pearson product-moment correlation analysis to explore the relationships between each variable. Each dimension of the family environment underwent separate analysis for indirect associations. To perform the data analysis, SPSS and the PROCESS computational macro were employed, facilitating indirect association and moderation analyses within SPSS. Additionally, the indirect association model was analyzed using AMOS. To ensure the robustness of the indirect association effects, a bootstrapping procedure with 5,000 bootstrap samples was employed to estimate the 95% confidence intervals.Control variables such as age, gender, and grade were carefully included in the model as covariates to account for potential confounding factors. Significance assessment was conducted using two-tailed tests, with a p-value set at 0.05.Furthermore, it is important to note that all analyses were conducted using SPSS 24.0 for Windows, ensuring standardized and reliable data processing.

## 3 Results

### 3.1 Demographic characteristics

In the survey, 6296 pairs of adolescents and parents took part. Among the adolescents, 3412 (54.2%) of them were male, and 2884 (45.8%) of them were female, with an average age of 13.45 years (SD=2.07). There are 650 individuals addicted to mobile and electronic products, with a detection rate of 10.3% for mobile and electronic product addiction. **Table 1** classified parents into groups based on their anxiety levels and compared their differences in family environment, emotional and behavioral issues of adolescents, and internet addiction. The analysis revealed significant variations among the groups, indicating the potential impact of parental anxiety on these aspects.

**Table 1:** Analysis of differences among three groups of parents: no anxiety, mild anxiety, and moderate to severe anxiety.

	No anxiety (n=5698)		Mild anxiety (n=574)		Moderate to severe anxiety (n=24)		F	*P*
	Mean	SD	Mean	SD	Mean	SD		
EBP	10.24	5.90	12.80	6.63	13.92	8.64	12.25	0.00
INT	4.92	3.24	6.21	3.67	6.42	4.60	10.86	0.00
EXT	7.51	4.10	9.19	4.73	9.75	6.00	11.35	0.00
Intimacy	7.66	1.89	6.92	2.36	6.13	2.98	-10.95	0.00
Entertainment	5.09	2.24	4.42	2.26	4.67	2.37	-7.92	0.00
Achievement	4.57	1.40	4.36	1.52	4.17	1.40	-4.16	0.00
Control	3.39	2.00	3.47	1.96	3.08	1.67	0.50	0.62
Cultural	4.95	2.18	4.41	2.15	3.83	2.30	-7.02	0.00
Conflicts	2.26	1.95	2.96	2.26	3.54	2.59	9.64	0.00
Organization	6.53	1.93	5.92	2.13	5.21	2.47	-9.12	0.00
IA	42.27	16.41	48.68	19.19	52.50	23.97	11.22	0.00

EBP, emotional and behavioral problem; INT, internalizing problems; EXT, externalizing problems; IA, Internet addiction; intimacy, entertainment, achievement, control, cultural, conflicts, organization are all dimensions of the family environment.

### 3.2 Preliminary correlation analyses

The results reveal significant positive correlations between parental anxiety, adolescent emotional and behavioral issues, and adolescent internet addiction variables (**Table 2**). These three factors show negative correlations with family environment’s intimacy, entertainment, achievement, culture, and organization, while they exhibit positive correlations with its control and conflict. These correlations align with our expectations, indicating support for our hypotheses that there is a close association between parental anxiety, adolescent emotional and behavioral issues, and adolescent internet addiction. Furthermore, these correlations are statistically significant in some instances, further emphasizing their importance and influence. It is worth noting that no significant correlation was found between parental anxiety and adolescent emotional and behavioral issues in the dimension of family environment control.

**Table 2:** Correlations between parental anxiety, family environment, teenage emotional and behavioral issues, and internet addiction.

Variables	Mean	SD	1	2	3	4	5	6	7	8	9
1 parent anxiety	1.06	2.432	–	–	–	–	–	–	–	–	–
2 EBP	10.48	6.031	0.153***	–	–	–	–	–	–	–	–
3 intimacy	7.58	1.958	-0.137***	-0.556***	–	–	–	–	–	–	–
4 entertainment	5.02	2.254	-0.100***	-0.394***	–	–	–	–	–	–	–
5 achievement	4.55	1.414	-0.052***	-0.123***	–	–	–	–	–	–	–
6 control	3.4	1.997	0.006	0.019	–	–	–	–	–	–	–
7 cultural	4.89	2.188	-0.088***	-0.359***	–	–	–	–	–	–	–
8 conflicts	2.33	1.992	0.121***	0.520***	–	–	–	–	–	–	–
9 organization	6.47	1.957	-0.114***	-0.493***	–	–	–	–	–	–	–
10 IA	42.89	16.827	0.140***	0.554***	-0.378***	-0.327***	-0.127***	-0.045***	-0.383***	0.338***	-0.389***

EBP, emotional and behavioral problem; INT, internalizing problems; EXT, externalizing problems; IA, Internet addiction; intimacy, entertainment, achievement, control, cultural, conflicts, organization are all dimensions of the family environment, ***p<0.001.

### 3.3 Indirect relationship analysis

**Figure 1:**
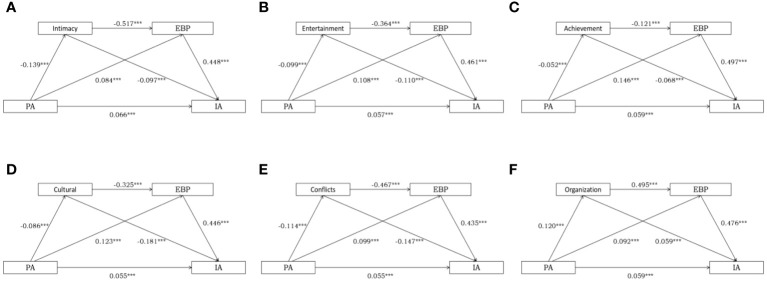
The relationship of family environment and children’s emotional/behavioral difficulties in parental anxiety and adolescent internet addiction. **(A-F)** represent the model between parental anxiety (PA) and Internet addiction (IA) through family environment (intimacy, entertainment, achievement, cultural, conflicts, organization) and adolescent emotional and behavioral problems (EBP), respectively.

We conducted an analysis of indirect relationships using regression analysis and bootstrapping, following Hayes’ (65)method, to examine the indirect influence of various dimensions of the family environment and emotional behavioral issues in adolescents on the relationship between parental anxiety and internet addiction. The results of the analysis are presented in **Table 3** and **Figure 1**. After accounting for sociodemographic variables (age, gender, grade), the direct effects, indirect effects, and total effects of the model were found to be statistically significant. Additionally, the non-parametric bootstrapping method confirmed the statistical significance of the indirect effects of each factor.

**Table 3:** Analysis of the pathway.

The pathway		Effect	SE	BootLLCI	BootULCI	% of contribution
Intimacy	Total effect	0.9690	0.0863	0.7997	1.1382 *	
	Direct effect	0.3573	0.0731	0.2140	0.5005 *	36.87%
	Ind1	0.0916	0.0178	0.0595	0.1281 *	9.45%
	Ind2	0.2661	0.0405	0.1888	0.3471 *	27.46%
	Ind3	0.2540	0.0310	0.1956	0.3167 *	26.21%
Entertainment	Total effect	0.9452	0.0879	0.7729	1.1175 *	
	Direct effect	0.3601	0.0740	0.2149	0.5052 *	38.10%
	Ind1	0.0890	0.0145	0.0624	0.1192 *	9.42%
	Ind2	0.3640	0.0473	0.2763	0.4613 *	38.51%
	Ind3	0.1320	0.0183	0.0970	0.1680 *	13.97%
Achievement	Total effect	0.9707	0.0863	0.8016	1.1398 *	
	Direct effect	0.3812	0.0731	0.2378	0.5245 *	39.27%
	Ind1	0.0210	0.0069	0.0090	0.0362 *	2.16%
	Ind2	0.5460	0.0562	0.4366	0.6571 *	56.25%
	Ind3	0.0225	0.0064	0.0105	0.0357 *	2.32%
Control	Total effect	0.9690	0.0863	0.7997	1.1382 *	
	Direct effect	0.3942	0.0731	0.2509	0.5375 *	40.68%
	Ind1	-0.0024	0.0047	-0.0121	0.0063	-0.25%
	Ind2	0.5768	0.0582	0.4643	0.6934 *	59.53%
	Ind3	0.0004	0.0010	-0.0014	0.0028	0.04%
Cultural	Total effect	0.9690	0.0863	0.7997	1.1382 *	
	Direct effect	0.3434	0.0713	0.2036	0.4831 *	35.44%
	Ind1	0.1274	0.0189	0.0919	0.1657 *	13.15%
	Ind2	0.3979	0.0463	0.3091	0.4911 *	41.06%
	Ind3	0.1002	0.0146	0.0726	0.1295 *	10.34%
Conflicts	Total effect	0.9690	0.0863	0.7997	1.1382 *	
	Direct effect	0.3737	0.0732	0.2302	0.5172 *	38.57%
	Ind1	0.0546	0.0131	0.0304	0.0820 *	5.63%
	Ind2	0.3228	0.0460	0.2365	0.4125 *	33.31%
	Ind3	0.2178	0.0268	0.1658	0.2715 *	22.48%
Organization	Total effect	0.9690	0.0863	0.7997	1.1382 *	
	Direct effect	0.3514	0.0724	0.2094	0.4934 *	36.26%
	Ind1	0.1185	0.0180	0.0848	0.1557 *	12.23%
	Ind2	0.3190	0.0424	0.2373	0.4061 *	32.92%
	Ind3	0.1800	0.0227	0.1358	0.2249 *	18.58%

PA, parent anxiety; EBP, emotional and behavioral problem; IA, Internet addiction. Taking Intimacy as an example, Ind1 is PA-Intimacy-IA, Ind2 is PA-EBP-IA, Ind3 is PA-Intimacy-EBP-IA. Similarly, for other dimensions and so forth.

SE stands for Standard Error, BootLLCI and BootUPCI are the lower and upper limit confidence intervals obtained through the Bootstrap method, the asterisk (*) represents significance in that pathway.

The relationship analysis regarding family environment intimacy and adolescent emotional/behavioral issues revealed significant effects between parental anxiety and adolescent internet addiction. The calculated indirect effect was 0.3573, with a confidence interval of [0.2140-0.5005], excluding 0, accounting for 36.87% of the total effect (0.9690) of parental anxiety on adolescent mobile internet addiction. Importantly, the indirect effect comprises three pathways, all of which are statistically significant. The first pathway, facilitated by parental anxiety - family environment intimacy - adolescent internet addiction, accounted for 9.45% of the total effect, with a confidence interval of [0.0595-0.1281]. The second pathway, influenced by parental anxiety - adolescent emotional/behavioral issues - adolescent internet addiction, accounted for 27.46% of the total effect, with a confidence interval of [0.1888-0.3471]. Lastly, the third pathway, involving parental anxiety - family environment intimacy - adolescent emotional/behavioral issues - adolescent internet addiction, accounted for 26.21% of the total effect, with a confidence interval of [0.1956-0.3167]. These findings indicate that family environment intimacy and adolescent emotional/behavioral issues play an indirect role in the connection between parental anxiety and adolescent internet addiction.

Moreover, the findings for other family environment dimensions, such as entertainment, achievement, cultural, and organizational aspects, exhibited a similar negative correlation with parental anxiety. In contrast, family environment conflict displayed a positive correlation with parental anxiety. These findings corroborate our initial hypothesis that family environment intimacy, entertainment, achievement, cultural, conflict, and organizational aspects, along with adolescent emotional and behavioral problems, may play a role in the association between parental anxiety and adolescent internet addiction. We also compared the overall magnitude of indirect effects for each model (see **Table 3**). Given that we compared the influence of family dimensions across models, and the confidence intervals for ind1 and ind3 of the family environment control model encompassed 0, we excluded it from consideration. We observed that family environment achievement had the largest magnitude of indirect effects, suggesting that adolescent emotional and behavioral problems and family environment achievement may have the greatest impact on the relationship between parental anxiety and adolescent internet addiction.

## 4 Discussion

The aim of this study was to examine the indirect impact of parental anxiety on internet addiction in adolescents. The results indicated a significant statistical association between parental anxiety and internet addiction through the indirect relationships of different dimensions of family environment and adolescent emotional and behavioral problems. These findings support the perspective of Family Systems Theory (66, 67), which posits that the behavior of each family member is influenced by others and, in turn, also affects the behaviors of other members within the family. This helps explain how parental anxiety may lead to internet addiction through the indirect relationships of different dimensions of family environment and adolescent emotional and behavioral problems. Among these factors, family environment intimacy seems to play a key role in influencing adolescent internet addiction. It is crucial for parents and adolescents to recognize the importance of family environment and work together to maintain a positive and beneficial family atmosphere. These results will be further discussed.

### 4.1 Correlation between parental anxiety and internet addiction

Firstly, in the data analysis of internet addiction prevalence, we paid particular attention to the differences in prevalence rates across various studies and found that this may be related to the methods of questionnaire administration and the characteristics of the study population. Some studies administered questionnaires through e-commerce websites such as JD.com, as well as social media platforms like WeChat and Weibo. This approach may not have adequately excluded participants who frequently use the internet in their daily lives, potentially leading to an overestimation of internet addiction prevalence in the results. We chose to conduct our survey in computer rooms at multiple schools, covering students from various grades, to enhance the credibility of the data and observe the general level of the study population. In our study, we found a significant association between parental anxiety and adolescent internet addiction in each model, which aligns with our previous hypotheses. Similar findings have been reported in previous research, indicating that parental anxiety is associated with various types of addictive behaviors, such as alcohol abuse (68), tobacco addiction (69), and food addiction (70). Some studies suggest that parents’ high levels of anxiety can impact the development of parental control, potentially influencing both internal and external issues in adolescents (71). The Ecological Systems Theory suggests that when the needs of children and parents are not aligned, parental attempts at psychological control of their children’s needs may lead to psychological imbalances, which in turn may result in emotional/behavioral problems and various substance addictions, especially internet addiction (72). Meanwhile, these relationships are considered to be partially mediated, implying that parental anxiety is not only directly associated with adolescent internet addiction but also indirectly influences adolescent internet addiction behavior through other variables (such as family environment intimacy). This study further illuminates the close association between parental anxiety and adolescent internet addiction, offering insights for a more in-depth understanding of this connection. It holds practical significance for the development of effective family support and educational programs, as well as the prevention and intervention of adolescent internet addiction behavior.

### 4.2 The effect of family environment

In line with our expectations, we found that parental anxiety not only directly influences internet addiction but also generates an indirect impact through the family environment. According to the Family Systems Theory, as a mutually interdependent entity, family environment can be affected by parental anxiety, thereby influencing the functioning and dynamics of the entire family system, which, in turn, may impact the child and make them vulnerable to substance dependence (73). In other words, in families where parental anxiety is present, the family environment undergoes negative changes, including increased instability, heightened conflicts, and reduced intimacy. These changes contribute to an escalation in adolescent emotional and behavioral problems, as well as an increase in internet addiction. A meta-analysis pointed out that when parents feel anxious, they may impose their own thoughts on their children in an abnormal way, intervening in their thoughts and behaviors, leading to a sense of insecurity and belongingness in children, which further contributes to a deteriorating family environment (74). Systemic relational approach emphasizes the interactions and influences among family members, highlighting the holistic and dynamic nature of the family system. In this approach, parental anxiety may disrupt the balance of the family system, affecting the stability and intimacy of internal family relationships, thereby negatively impacting the emotions and behaviors of adolescents (75). On the other hand, attachment theory focuses on the emotional bond between children and primary caregivers, suggesting that children’s attachment experiences influence their emotional and behavioral development (76). In families with anxious parents, issues related to insecure attachment with parents may exist, potentially leading to an increased need for intimate relationships among adolescents, thereby influencing their internet addiction behavior. Additionally, adolescents may experience stress and a lack of confidence when confronted with challenges, leading to a reduction in intimacy with family members. Research has revealed that this psychological state inclines adolescents to distance themselves from the family, making it challenging to establish profound emotional connections (77). Therefore, comprehending and supporting adolescents in times of adversity are crucial for maintaining family intimacy. Other research has also found a positive correlation between controlling family environment and adolescent frustration (78), and an increase in frustration can drive adolescents to seek refuge in the virtual world of the internet. Parental anxiety may lead to a poorer family environment, and the lack of security and belongingness may drive adolescents to seek the internet as a possible comfort, ultimately leading to internet addiction. Furthermore, entertainment-oriented and success-oriented characteristics may have positive effects on adolescents. A family environment rich in entertainment may encourage adolescents to seek healthy forms of entertainment, thereby reducing their reliance on the internet (79). Likewise, a success-oriented family may instill positive values of achievement, encouraging adolescents to pursue goals through tangible actions, thereby reducing reliance on the internet as a crutch (80). However, these positive influences may be weakened or even offset when there is conflict and chaos within the family environment. Internal family conflicts may lead adolescents to feel stressed and insecure, making them more prone to seeking ways to escape reality, such as getting lost in the internet (81). The disorderliness of family organization may weaken intimacy among family members, leaving adolescents feeling lonely and unstable, thus increasing the risk of internet addiction (82). On the other hand, family culture also influences adolescent internet addiction. A positive, healthy family culture may emphasize communication, mutual support, and personal development, providing a more conducive environment for adolescents’ healthy growth. Conversely, a lack of clear values and cultural heritage in the family may lead to confusion and a lack of self-worth in adolescents, making them more susceptible to seeking solace in virtual worlds like the internet (83). In other words, parental anxiety has an indirect relationship with internet addiction in middle and high school students, with the family environment being the influencing factor. However, the potential lack of significance in the indirect pathways in the control model may be attributed to various complex factors. Firstly, the influence of family environment control on adolescent emotional and behavioral issues may be subject to moderation or interference from other factors, thereby diminishing the pathway’s significance. Secondly, while family environment control acts as a mediating variable transmitting parental anxiety’s impact on adolescent internet addiction, there could be other more substantial mediating factors overlooked in the current model. Additionally, other important factors such as individual characteristics of adolescents, social environment, mental health status, and socioeconomic status, which were not taken into account, may play pivotal roles in this relationship.

### 4.3 The effect of adolescent emotional and behavioral problems

Furthermore, parental anxiety also directly and indirectly predicted internet addiction through adolescent emotional and behavioral problems. In each model, the contribution of Ind2 is relatively high, suggesting that parental anxiety may directly affect adolescent emotional and behavioral issues, while also potentially indirectly influencing adolescents’ tendency towards internet addiction through its impact on the family environment. The significant contribution of this pathway highlights the crucial influence of parental emotional state on both the family atmosphere and the behavioral development of adolescents. Studies have shown that adolescent depression symptoms are associated with genetic and environmental risk factors (84). Parental anxiety may lead to excessive expectations and attention towards their children’s academic performance. This anxiety may stem from concerns about their children’s future, fearing that they may not meet the standards set by the parents, thus affecting their future academic achievements and life development. Consequently, parents may attempt to address this anxiety by emphasizing and expecting their children’s academic performance, ensuring that they achieve success and fulfill the desired accomplishments (85). A research (86) indicated that under the backdrop of COVID-19, parents with higher levels of anxiety and depression displayed worsened attitudes towards their children, which was closely associated with the increase in children’s psychological problems. Conversely, in the family setting, if emotional support is high and conflicts are low, children and adolescents are more likely to feel protected, thereby reducing the incidence of emotional and behavioral problems (87). Therefore, maintaining positive and intimate relationships among family members is crucial. Studies on internet use have found a close association between internet addiction and psychological issues (88). A study on Singaporean university students found a significant correlation between emotional disorders and addictive behaviors, particularly in females (89). A meta-analysis indicates that during the pandemic, the impact of internet addiction on emotional issues has intensified (90). This could be attributed to the implementation of global social distancing and isolation policies, leading many individuals to experience isolation and anxiety. In such an environment, internet addiction may serve as a means for people to escape negative emotions, further exacerbating the association with emotional problems. Consequently, parental anxiety may contribute to emotional and behavioral issues in adolescents, prompting them to seek solace through the internet, ultimately resulting in internet addiction.This study deepens our understanding of the relationships between parental anxiety, family environment, adolescent emotional and behavioral issues, and internet addiction, providing substantive guidance for the development of effective family support and preventive intervention plans.

### 4.4 The multiple relationship model

Our research findings indicate that adolescent emotional and behavioral problems and family environment play an indirect role in the relationship between parental anxiety and adolescent internet addiction. Previous studies have shown that parental anxiety may be transmitted to adolescents through parenting styles or by imitating parental behaviors, leading to emotional and behavioral problems in adolescents (91). Sell et al. (92) further found a close association between family environment and adolescent emotional and behavioral problems. Moreover, the results show that adolescent emotional and behavioral problems are negatively correlated with the intimacy, recreation, achievement, cultural orientation, and organization of the family environment, while positively correlated with conflicts. The above discussions suggest that parental anxiety is related to family environment, and adolescent emotional and behavioral problems are associated with internet addiction. Therefore, parental anxiety may lead to a poorer family environment, which, in turn, may cause emotional and behavioral problems in children, ultimately leading to internet addiction. In future research, we will integrate additional studies to provide targeted guidance aimed at improving family environments and addressing adolescent emotional and behavioral issues as well as internet addiction.

### 4.5 Limitation

There are several limitations in the current study. Firstly, the proportion of mothers in the sample was higher than fathers, with a ratio of approximately 2:1, leading to an imbalance in parental representation, making it difficult to distinguish the effects of each parent on adolescents. Mothers often serve as the primary caregivers for adolescents, which may contribute to this imbalance. However, further research is needed to delve deeper into the anxiety levels of fathers to comprehensively understand the influence of both parents on adolescents’ development. Secondly, although the Bootstrap method was used, the cross-sectional design may not elucidate the causal relationship between parental anxiety and internet addiction and may lead to biased parameter estimates (93). Therefore, longitudinal studies are needed to further establish causality. Furthermore, there are certain limitations to the generalizability of this study, as our sample primarily consists of adolescents from middle school, high school, and elementary school, and the data is from Jiangsu Province, China. Due to the specificity of the sample, the generalization of research findings may be influenced by cultural or regional differences. Therefore, when extrapolating the study results, it is crucial to consider the unique background of this particular group to ensure broader applicability of the research. Finally, despite achievement having the highest coefficient in the family environment structure, we did not include it in the model when considering the variance of other family environment structures. Therefore, we plan to employ an incremental prediction model in future research to further explore this area.

## 5 Conclusion

This study investigated 6,296 parent-child pairs to delve into the intricate relationship between parental anxiety and adolescent internet addiction, while also examining the impact of child emotional and behavioral problems and family environment. Parental anxiety not only directly affects adolescent internet addiction but also indirectly influences it through factors such as family environment and child emotional and behavioral problems. This discovery underscores the significance of addressing parental anxiety proactively and nurturing a supportive family environment. In terms of family environment, our study investigated different dimensions of family dynamics. Therefore, our research not only contributes to understanding the overall impact of family factors on adolescent internet addiction but also provides specific insights into how various family constructs influence internet addiction. In future research, we will further explore the association between parenting dimensions and styles with externalizing problems in children and adolescents, in order to develop more targeted and scientifically effective intervention measures. This will not only help address emotional and behavioral issues in adolescents, but also aid in the prevention and treatment of internet addiction. In today’s information age, the mental well-being of adolescents is of paramount importance, therefore, this study provides valuable insights into family dynamics, parent-child relationships, and mental health for society, offering a robust foundation for future policies and interventions.

## Data availability statement

The original contributions presented in the study are included in the article/supplementary material. Further inquiries can be directed to the corresponding authors.

## Ethics statement

The studies involving humans were approved by Medical Research Ethics Committee of the Affiliated Brain Hospital of Nanjing Medical University. The studies were conducted in accordance with the local legislation and institutional requirements. Written informed consent for participation in this study was provided by the participants’ legal guardians/next of kin.

## Author contributions

YuW: Writing – original draft, Writing – review & editing. KZ: Formal analysis, Writing – review & editing. YaW: Methodology, Supervision, Writing – review & editing. JZ: Writing – review & editing. YX: Writing – review & editing. XW: Writing – review & editing. WY: Writing – review & editing. XZ: Writing – review & editing. JY: Writing – review & editing. FW: Data curation, Funding acquisition, Investigation, Project administration, Supervision, Writing – review & editing.

## References

[1] Wakefield JC. Diagnostic issues and controversies in DSM-5: return of the false positives problem. Annu Rev Clin Psychol. (2016) 12:105–32. doi: 10.1146/annurev-clinpsy-032814-112800 26772207

[2] Gjoneska B Potenza MN Jones J Corazza O Hall N Sales CMD. Problematic use of the internet during the COVID-19 pandemic: Good practices and mental health recommendations. Compr Psychiatry. (2022) 112:152279. doi: 10.1016/j.comppsych.2021.152279 34700188 PMC8529894

[3] Vasudevan K Fung DSS. Editorial: internet addiction & Gaming disorders in children and adolescents. Front Psychiatry. (2022) 13:870177. doi: 10.3389/fpsyt.2022.870177 35370844 PMC8964974

[4] Wölfling K Müller KW Dreier M Ruckes C Deuster O Batra A. Efficacy of short-term treatment of internet and computer game addiction: A randomized clinical trial. JAMA Psychiatry. (2019) 76:1018–25. doi: 10.1001/jamapsychiatry.2019.1676 PMC6624826 31290948

[5] Shehata WM Abdeldaim DE. Internet addiction among medical and non-medical students during COVID-19 pandemic, Tanta University, Egypt. Environ Sci pollut Res Int. (2021) 28:59945–52. doi: 10.1007/s11356-021-14961-9 PMC8214711 34148197

[6] Jiang Q Chen Z Zhang Z Zuo C. Investigating links between Internet literacy, Internet use, and Internet addiction among Chinese youth and adolescents in the digital age. Front Psychiatry. (2023) 14:1233303. doi: 10.3389/fpsyt.2023.1233303 37743978 PMC10513100

[7] Ran G Li J Zhang Q Niu X. The association between social anxiety and mobile phone addiction: A three-level meta-analysis. Comput Hum Behav. (2022) 130:N.PAG–G. doi: 10.1016/j.chb.2022.107198

[8] Bhandari PM Neupane D Rijal S Thapa K Mishra SR Poudyal AK. Sleep quality, internet addiction and depressive symptoms among undergraduate students in Nepal. BMC Psychiatry. (2017) 17:106. doi: 10.1186/s12888-017-1275-5 28327098 PMC5361804

[9] Al-Khani AM Saquib J Rajab AM Khalifa MA Almazrou A Saquib N. Internet addiction in Gulf countries: A systematic review and meta-analysis. J Behav Addict. (2021) 10:601–10. doi: 10.1556/2006.2021.00057 PMC8997198 34491902

[10] Cai J Wang Y Wang F Lu J Li L Zhou X. The association of parent-child communication with internet addiction in left-behind children in China: A cross-sectional study. Int J Public Health. (2021) 66:630700. doi: 10.3389/ijph.2021.630700 34744584 PMC8565268

[11] Zhu X Deng C Bai W. Parental control and adolescent internet addiction: the moderating effect of parent-child relationships. Front Public Health. (2023) 11:1190534. doi: 10.3389/fpubh.2023.1190534 37304126 PMC10248257

[12] Cheng C Lau YC Chan L Luk JW. Prevalence of social media addiction across 32 nations: Meta-analysis with subgroup analysis of classification schemes and cultural values. Addictive Behav. (2021) 117:106845. doi: 10.1016/j.addbeh.2021.106845 33550200

[13] Tang X Lu Z Hu D Zhong X. Influencing factors for prenatal Stress, anxiety and depression in early pregnancy among women in Chongqing, China. J Affect Disord. (2019) 253:292–302. doi: 10.1016/j.jad.2019.05.003 31077972

[14] Li X Li D Newman J. Parental behavioral and psychological control and problematic internet use among Chinese adolescents: the mediating role of self-control. Cyberpsychol Behav Soc Network. (2013) 16:442–7. doi: 10.1089/cyber.2012.0293 23509987

[15] Shek DTL Zhu X Ma CMS. The influence of parental control and parent-child relational qualities on adolescent internet addiction: A 3-year longitudinal study in Hong Kong. Front Psychol. (2018) 9:642. doi: 10.3389/fpsyg.2018.00642 29765349 PMC5938405

[16] Wang D Nie X Zhang D Hu Y. The relationship between parental psychological control and problematic smartphone use in early Chinese adolescence: A repeated-measures study at two time-points. Addictive Behav. (2022) 125:107142. doi: 10.1016/j.addbeh.2021.107142 34673361

[17] Yu Y Mo PK Zhang J Li J Lau JT. Impulsivity, self-control, interpersonal influences, and maladaptive cognitions as factors of internet gaming disorder among adolescents in China: cross-sectional mediation study. J Med Internet Res. (2021) 23:e26810. doi: 10.2196/26810 34704960 PMC8581749

[18] Kwak JY Kim JY Yoon YW. Effect of parental neglect on smartphone addiction in adolescents in South Korea. Child Abuse Neglect. (2018) 77:75–84. doi: 10.1016/j.chiabu.2017.12.008 29306184

[19] Stein A Pearson RM Goodman SH Rapa E Rahman A McCallum M. Effects of perinatal mental disorders on the fetus and child. Lancet (London England). (2014) 384:1800–19. doi: 10.1016/S0140-6736(14)61277-0 25455250

[20] Woody ML Kaurin A McKone KMP Ladouceur CD Silk JS. Displays of negative facial affect during parent-adolescent conflict and the bidirectional transmission of social anxiety. J Child Psychol Psychiatry. (2022) 63:846–54. doi: 10.1111/jcpp.13530 PMC8986877 34617605

[21] Aktar E Nikolić M Bögels SM. Environmental transmission of generalized anxiety disorder from parents to children: worries, experiential avoidance, and intolerance of uncertainty. Dialogues Clin Neurosci. (2017) 19:137–47. doi: 10.31887/DCNS.2017.19.2/eaktar PMC5573558 28867938

[22] Luebbe AM Tu C Fredrick JW. Socialization goals, parental psychological control, and youth anxiety in Chinese students: moderated indirect effects based on school type. J Youth Adolesc. (2018) 47:413–29. doi: 10.1007/s10964-017-0784-3 29110254

[23] Deater-Deckard K. Parents’ and children’s ADHD in a family system. J Abnormal Child Psychol. (2017) 45:519–25. doi: 10.1007/s10802-017-0276-7 28181060

[24] Nakao K Takaishi J Tatsuta K Katayama H Iwase M Yorifuji K. The influences of family environment on personality traits. Psychiatry Clin Neurosci. (2000) 54:91–5. doi: 10.1046/j.1440-1819.2000.00642.x 15558885

[25] Kininmonth AR Smith AD Llewellyn CH Dye L Lawton CL Fildes A. The relationship between the home environment and child adiposity: a systematic review. Int J Behav Nutr Phys Activity. (2021) 18:4. doi: 10.1186/s12966-020-01073-9 PMC7788808 33407598

[26] Larrucea-Iruretagoyena M Orue I. The mediating role of mindful parenting in the relationship between parental anxiety and youth’s emotional and behavioral difficulties. J Youth Adolesc. (2023) 52:1471–80. doi: 10.1007/s10964-023-01752-3 PMC10175410 36811698

[27] Li Y Li G Liu L Wu H. Correlations between mobile phone addiction and anxiety, depression, impulsivity, and poor sleep quality among college students: A systematic review and meta-analysis. J Behav Addict. (2020) 9:551–71. doi: 10.1556/2006.2020.00057 PMC8943681 32903205

[28] Zhao M Huang Y Wang J Feng J Zhou B. Internet addiction and depression among Chinese adolescents: anxiety as a mediator and social support as a moderator. Psychol Health Med. (2023) 28:2315–28. doi: 10.1080/13548506.2023.2224041 37317485

[29] Wang ES Wang MC. Social support and social interaction ties on internet addiction: integrating online and offline contexts. Cyberpsychol Behav Soc Network. (2013) 16:843–9. doi: 10.1089/cyber.2012.0557 23848959

[30] Clark AT Arnoldi JF Zelnik YR Barabas G Hodapp D Karakoç C. General statistical scaling laws for stability in ecological systems. Ecol Lett. (2021) 24:1474–86. doi: 10.1111/ele.13760 33945663

[31] Emlen ST. An evolutionary theory of the family. Proc Natl Acad Sci U S A. (1995) 92:8092–9. doi: 10.1073/pnas.92.18.8092 PMC41102 7667250

[32] Lewis AJ. Attachment-based family therapy for adolescent substance use: A move to the level of systems. Front Psychiatry. (2019) 10:948. doi: 10.3389/fpsyt.2019.00948 32116807 PMC7025563

[33] Pérez-Edgar K. Editorial: moments in history as a catalyst for science: placing the individual within a specific time and place. J Am Acad Child Adolesc Psychiatry. (2021) 60:1185–6. doi: 10.1016/j.jaac.2021.03.006 33741475

[34] Peng L Hu R Feng Y Shi W Zhao L Jiang L. The relationship between family diet consumption, family environment, parent anxiety and nutrition status children during the COVID-19 pandemic: a longitudinal study. Front Public Health. (2023) 11:1228626. doi: 10.3389/fpubh.2023.1228626 37637798 PMC10447892

[35] Hong JC Juan HC Hung WC. The role of family intimacy in playing collaborative e-sports with a Switch device to predict the experience of flow and anxiety during COVID-19 lockdown. Comput Hum Behav. (2022) 132:107244. doi: 10.1016/j.chb.2022.107244 PMC8883355 35250161

[36] Nash SG McQueen A Bray JH. Pathways to adolescent alcohol use: family environment, peer influence, and parental expectations. J Adolesc Health. (2005) 37:19–28. doi: 10.1016/j.jadohealth.2004.06.004 15963903

[37] Shah J Das P Muthiah N Milanaik R. New age technology and social media: adolescent psychosocial implications and the need for protective measures. Curr Opin Pediatr. (2019) 31:148–56. doi: 10.1097/MOP.0000000000000714 30507648

[38] Yu CCW Wong SWL Lo FSF So RCH Chan DFY. Study protocol: a randomized controlled trial study on the effect of a game-based exercise training program on promoting physical fitness and mental health in children with autism spectrum disorder. BMC Psychiatry. (2018) 18(1):56. 29486750 10.1186/s12888-018-1635-9 PMC5830347

[39] Carman KL Dardess P Maurer M Sofaer S Adams K Bechtel C. Patient and family engagement: a framework for understanding the elements and developing interventions and policies. Health Aff (Millwood). (2013) 32:223–31. doi: 10.1377/hlthaff.2012.1133 23381514

[40] Lian SL Cao XX Xiao QL Zhu XW Yang C Liu QQ. Family cohesion and adaptability reduces mobile phone addiction: the mediating and moderating roles of automatic thoughts and peer attachment. Front Psychol. (2023) 14:1122943. doi: 10.3389/fpsyg.2023.1122943 37397308 PMC10311501

[41] Shah R Chauhan N Gupta AK Sen MS. Adolescent-parent conflict in the age of social media: Case reports from India. Asian J Psychiatr. (2016) 23:24–6. doi: 10.1016/j.ajp.2016.07.002 27969073

[42] Boz C Dinç M. Examination of game addiction studies conducted in Turkey: A systematic review study. Front Psychiatry. (2023) 14:1014621. doi: 10.3389/fpsyt.2023.1014621 37124255 PMC10140370

[43] Lozano-Blasco R Barreiro-Collazo A Romero-Gonzalez B Soto-Sanchez A. The family context in cybervictimization: A systematic review and meta-analysis. Trauma Violence Abuse. (2023), 15248380231207894. doi: 10.1177/15248380231207894 37947083

[44] Schneider LA King DL Delfabbro PH. Family factors in adolescent problematic Internet gaming: A systematic review. J Behav Addict. (2017) 6:321–33. doi: 10.1556/2006.6.2017.035 PMC5700711 28762279

[45] King DL Delfabbro PH Zwaans T Kaptsis D. Clinical features and axis I comorbidity of Australian adolescent pathological Internet and video game users. Aust New Z J Psychiatry. (2013) 47:1058–67. doi: 10.1177/0004867413491159 23719181

[46] Wang BQ Yao NQ Zhou X Liu J Lv ZT. The association between attention deficit/hyperactivity disorder and internet addiction: a systematic review and meta-analysis. BMC Psychiatry. (2017) 17:260. doi: 10.1186/s12888-017-1408-x 28724403 PMC5517818

[47] Atoum M Alhussami M Rayan A. Emotional and behavioral problems among Jordanian adolescents: Prevalence and associations with academic achievement. J Child Adolesc Psychiatr Nurs. (2018) 31:70–8. doi: 10.1111/jcap.12211 30298603

[48] Marin MG Nuñez X de Almeida RMM. Internet addiction and attention in adolescents: A systematic review. Cyberpsychol Behav Soc Network. (2021) 24:237–49. doi: 10.1089/cyber.2019.0698 33121255

[49] Rachubińska K Cybulska AM Grochans E. The relationship between loneliness, depression, internet and social media addiction among young Polish women. Eur Rev Med Pharmacol Sci. (2021) 25:1982–9. doi: 10.26355/eurrev_202102_25099 33660809

[50] Kaiser RH Snyder HR Goer F Clegg R Ironside M Pizzagalli DA. Attention bias in rumination and depression: cognitive mechanisms and brain networks. Clin Psychol Sci. (2018) 6:765–82. doi: 10.1177/2167702618797935 PMC6519952 31106040

[51] Lin YN Iao LS Lee YH Wu CC. Parenting stress and child behavior problems in young children with autism spectrum disorder: transactional relations across time. J Autism Dev Disord. (2021) 51:2381–91. doi: 10.1007/s10803-020-04720-z 32965625

[52] Shalev A Merranko J Goldstein T Miklowitz DJ Axelson D Goldstein BI. A longitudinal study of family functioning in offspring of parents diagnosed with bipolar disorder. J Am Acad Child Adolesc Psychiatry. (2019) 58:961–70. doi: 10.1016/j.jaac.2018.10.011 PMC6584080 30768400

[53] Repetti RL Taylor SE Seeman TE. Risky families: family social environments and the mental and physical health of offspring. psychol Bull. (2002) 128:330–66. doi: 10.1037/0033-2909.128.2.230 11931522

[54] Caster JB Inderbitzen HM Hope D. Relationship between youth and parent perceptions of family environment and social anxiety. J Anxiety Disord. (1999) 13:237–51. doi: 10.1016/S0887-6185(99)00002-X 10372340

[55] Goodman R. The Strengths and Difficulties Questionnaire: a research note. J Child Psychol Psychiatry Allied Disciplines. (1997) 38:581–6. doi: 10.1111/j.1469-7610.1997.tb01545.x 9255702

[56] Lai KY Luk ES Leung PW Wong AS Law L Ho K. Validation of the Chinese version of the strengths and difficulties questionnaire in Hong Kong. Soc Psychiatry Psychiatr Epidemiol. (2010) 45:1179–86. doi: 10.1007/s00127-009-0152-z 19820885

[57] Lee S Shin A. Association of atopic dermatitis with depressive symptoms and suicidal behaviors among adolescents in Korea: the 2013 Korean Youth Risk Behavior Survey. BMC Psychiatry. (2017) 17:3. doi: 10.1186/s12888-016-1160-7 28049449 PMC5209888

[58] Ko CH Yen JY Yen CF Chen CC Yen CN Chen SH. Screening for Internet addiction: an empirical study on cut-off points for the Chen Internet Addiction Scale. Kaohsiung J Med Sci. (2005) 21:545–51. doi: 10.1016/S1607-551X(09)70206-2 PMC11918109 16670046

[59] Lin MP Ko HC Wu JY. Prevalence and psychosocial risk factors associated with internet addiction in a nationally representative sample of college students in Taiwan. Cyberpsychol Behav Soc Network. (2011) 14:741–6. doi: 10.1089/cyber.2010.0574 21651418

[60] Marwaha S Palmer E Suppes T Cons E Young AH Upthegrove R. Novel and emerging treatments for major depression. Lancet (London England). (2023) 401:141–53. doi: 10.1016/S0140-6736(22)02080-3 36535295

[61] Risom SS Zwisler AD Johansen PP Sibilitz KL Lindschou J Gluud C. Exercise-based cardiac rehabilitation for adults with atrial fibrillation. Cochrane Database Syst Rev. (2017) 2:Cd011197. doi: 10.1002/14651858.CD011197.pub2 28181684 PMC6464537

[62] Yu Y Yang X Yang Y Chen L Qiu X Qiao Z. The role of family environment in depressive symptoms among university students: A large sample survey in China. PloS One. (2015) 10:e0143612. doi: 10.1371/journal.pone.0143612 26629694 PMC4667844

[63] Belardinelli C Hatch JP Olvera RL Fonseca M Caetano SC Nicoletti M. Family environment patterns in families with bipolar children. J Affect Disord. (2008) 107:299–305. doi: 10.1016/j.jad.2007.08.011 17905443

[64] Kroenke K Spitzer RL Williams JB Löwe B. The Patient Health Questionnaire Somatic, Anxiety, and Depressive Symptom Scales: a systematic review. Gen Hosp Psychiatry. (2010) 32:345–59. doi: 10.1016/j.genhosppsych.2010.03.006 20633738

[65] Bolin JH. Introduction to mediation, moderation, and conditional process analysis: A regression-based approach. J Educ Measure. (2014) 51:335–7. doi: 10.1111/jedm.12050

[66] Kaestle CE Allen KR Wesche R Grafsky EL. Adolescent sexual development: A family perspective. J Sex Res. (2021) 58:874–90. doi: 10.1080/00224499.2021.1924605 34003063

[67] Calatrava M Martins MV Schweer-Collins M Duch-Ceballos C Rodríguez-González M. Differentiation of self: A scoping review of Bowen Family Systems Theory’s core construct. Clin Psychol Rev. (2022) 91:102101. doi: 10.1016/j.cpr.2021.102101 34823190

[68] Kuntsche E Kuntsche S Thrul J Gmel G. Binge drinking: Health impact, prevalence, correlates and interventions. Psychol Health. (2017) 32:976–1017. doi: 10.1080/08870446.2017.1325889 28513195

[69] Spears CA Hedeker D Li L Wu C Anderson NK Houchins SC. Mechanisms underlying mindfulness-based addiction treatment versus cognitive behavioral therapy and usual care for smoking cessation. J Consult Clin Psychol. (2017) 85:1029–40. doi: 10.1037/ccp0000229 PMC5662477 28650195

[70] Hoover LV Yu HP Cummings JR Ferguson SG Gearhardt AN. Co-occurrence of food addiction, obesity, problematic substance use, and parental history of problematic alcohol use. Psychol Addictive Behav: J Soc Psychologists Addictive Behav. (2022) 37:928–35. doi: 10.1037/adb0000870 PMC10986778 35878078

[71] van der Bruggen CO Stams GJ Bögels SM. Research review: the relation between child and parent anxiety and parental control: a meta-analytic review. J Child Psychol Psychiatry Allied Disciplines. (2008) 49:1257–69. doi: 10.1111/j.1469-7610.2008.01898.x 18355216

[72] Wells MB Jeon L Aronson O. Bidirectional associations between paternal postpartum depression symptoms and coparenting: A cross-lagged panel model of fathers of infants and toddlers. J Affect Disord. (2023) 324:440–8. doi: 10.1016/j.jad.2022.12.128 36608849

[73] Pratt KJ Skelton JA. Family functioning and childhood obesity treatment: A family systems theory-informed approach. Acad Pediatr. (2018) 18:620–7. doi: 10.1016/j.acap.2018.04.001 PMC8111666 29654905

[74] Yap MB Jorm AF. Parental factors associated with childhood anxiety, depression, and internalizing problems: a systematic review and meta-analysis. J Affect Disord. (2015) 175:424–40. doi: 10.1016/j.jad.2015.01.050 25679197

[75] Feeney J Fitzgerald J. Attachment, conflict and relationship quality: laboratory-based and clinical insights. Curr Opin Psychol. (2019) 25:127–31. doi: 10.1016/j.copsyc.2018.04.002 29753972

[76] Bosmans G Van Vlierberghe L Bakermans-Kranenburg MJ Kobak R Hermans D van IMH. A learning theory approach to attachment theory: exploring clinical applications. Clin Child Fam Psychol Rev. (2022) 25:591–612. doi: 10.1007/s10567-021-00377-x 35098428 PMC8801239

[77] Wei S Teo T Malpique A Lausen A. Parental autonomy support, parental psychological control and Chinese university students’ Behavior regulation: the mediating role of basic psychological needs. Front Psychol. (2021) 12:735570. doi: 10.3389/fpsyg.2021.735570 35250687 PMC8895294

[78] El-Sheikh M Erath SA. Family conflict, autonomic nervous system functioning, and child adaptation: state of the science and future directions. Dev Psychopathol. (2011) 23:703–21. doi: 10.1017/S0954579411000034 PMC3695441 23786705

[79] O’Keeffe GS Clarke-Pearson K. The impact of social media on children, adolescents, and families. Pediatrics. (2011) 127:800–4. doi: 10.1542/peds.2011-0054 21444588

[80] Şenormancı Ö Şenormancı G Güçlü O Konkan R. Attachment and family functioning in patients with internet addiction. Gen Hosp Psychiatry. (2014) 36:203–7. doi: 10.1016/j.genhosppsych.2013.10.012 24262601

[81] Gao T Meng X Qin Z Zhang H Gao J Kong Y. Association between parental marital conflict and Internet addiction: A moderated mediation analysis. J Affect Disord. (2018) 240:27–32. doi: 10.1016/j.jad.2018.07.005 30048833

[82] Nakayama H Mihara S Higuchi S. Treatment and risk factors of Internet use disorders. Psychiatry Clin Neurosci. (2017) 71:492–505. doi: 10.1111/pcn.12493 27987253

[83] Chi X Lin L Zhang P. Internet addiction among college students in China: prevalence and psychosocial correlates. Cyberpsychol Behav Soc Netw. (2016) 19:567–73. doi: 10.1089/cyber.2016.0234 27635444

[84] Kwong ASF López-López JA Hammerton G Manley D Timpson NJ Leckie G. Genetic and environmental risk factors associated with trajectories of depression symptoms from adolescence to young adulthood. JAMA Netw Open. (2019) 2:e196587. doi: 10.1001/jamanetworkopen.2019.6587 31251383 PMC6604106

[85] Cao W Fang Z Hou G Han M Xu X Dong J. The psychological impact of the COVID-19 epidemic on college students in China. Psychiatry Res. (2020) 287:112934. doi: 10.1016/j.psychres.2020.112934 32229390 PMC7102633

[86] Brown SM Doom JR Lechuga-Peña S Watamura SE Koppels T. Stress and parenting during the global COVID-19 pandemic. Child Abuse Negl. (2020) 110:104699. doi: 10.1016/j.chiabu.2020.104699 32859394 PMC7440155

[87] Wathelet M Duhem S Vaiva G Baubet T Habran E Veerapa E. Factors associated with mental health disorders among university students in France confined during the COVID-19 pandemic. JAMA Netw Open. (2020) 3:e2025591. doi: 10.1001/jamanetworkopen.2020.25591 33095252 PMC7584927

[88] Pantic I. Online social networking and mental health. Cyberpsychol Behav Soc Network. (2014) 17:652–7. doi: 10.1089/cyber.2014.0070 PMC4183915 25192305

[89] Tang CS Koh YY. Online social networking addiction among college students in Singapore: Comorbidity with behavioral addiction and affective disorder. Asian J Psychiatry. (2017) 25:175–8. doi: 10.1016/j.ajp.2016.10.027 28262144

[90] Pallavicini F Pepe A Mantovani F. The effects of playing video games on stress, anxiety, depression, loneliness, and gaming disorder during the early stages of the COVID-19 pandemic: PRISMA systematic review. Cyberpsychol Behav Soc Network. (2022) 25:334–54. doi: 10.1089/cyber.2021.0252 35639118

[91] Ayano G Betts K Maravilla JC Alati R. The risk of anxiety disorders in children of parents with severe psychiatric disorders: a systematic review and meta-analysis. J Affect Disord. (2021) 282:472–87. doi: 10.1016/j.jad.2020.12.134 33422825

[92] Sell M Barkmann C Adema B Daubmann A Kilian R Stiawa M. Associations of family functioning and social support with psychopathology in children of mentally ill parents: multilevel analyses from different rating perspectives. Front Psychol. (2021) 12:705400. doi: 10.3389/fpsyg.2021.705400 34594270 PMC8476746

[93] Maxwell SE Cole DA. Bias in cross-sectional analyses of longitudinal mediation. psychol Methods. (2007) 12:23–44. doi: 10.1037/1082-989X.12.1.23 17402810

